# Oncolytic virus efficiency inhibited growth of tumour cells with multiple drug resistant phenotype in vivo and in vitro

**DOI:** 10.1186/s12967-016-1002-x

**Published:** 2016-08-18

**Authors:** Elena P. Goncharova, Julia S. Ruzhenkova, Ivan S. Petrov, Sergey N. Shchelkunov, Marina A. Zenkova

**Affiliations:** 1 Institute of Chemical Biology and Fundamental Medicine SB RAS, 8, Lavrentiev ave., Novosibirsk, 630090 Russian Federation; 2 Institute of Cytology and Genetics SB RAS, Novosibirsk, Russian Federation; 3 Department of Biochemistry, Biocenter, University of Wuerzburg, Am Hubland, 97074 Würzburg, Germany

**Keywords:** Multiple drug resistance, Vaccinia virus, Cancer, Human and murine cancer cells, Melanoma B16, Oncolytic action, Virotherapy, Interleukin IL-6, Immunotherapy

## Abstract

**Background:**

Tumour resistance to a wide range of drugs (multiple drug resistant, MDR) acquired after intensive chemotherapy is considered to be the main obstacle of the curative treatment of cancer patients. Recent work has shown that oncolytic viruses demonstrated prominent potential for effective treatment of diverse cancers. Here, we evaluated whether genetically modified vaccinia virus (LIVP-GFP) may be effective in treatment of cancers displaying MDR phenotype.

**Methods:**

LIVP-GFP replication, transgene expression and cytopathic effects were analysed in human cervical carcinomas KB-3-1 (MDR−), KB-8-5 (MDR+) and in murine melanoma B-16 (MDR−), murine lymphosarcomas RLS and RLS-40 (MDR+). To investigate the efficacy of this therapy in vivo, we treated immunocompetent mice bearing murine lymphosarcoma RLS-40 (MDR+) (6- to 8-week-old female CBA mice; n = 10/group) or melanoma B-16 (MDR−) (6- to 8-week-old female C57Bl mice; n = 6/group) with LIVP-GFP (5 × 10^7^ PFU of virus in 0.1 mL of IMDM immediately and 4 days after tumour implantation).

**Results:**

We demonstrated that LIVP-GFP replication was effective in human cervical carcinomas KB-3-1 (MDR−) and KB-8-5 (MDR+) and in murine melanoma B-16 (MDR−), whereas active viral production was not detected in murine lymphosarcomas RLS and RLS-40 (MDR+). Additionally, it was found that in tumour models in immunocompetent mice under the optimized regimen intratumoural injections of LIVP-GFP significantly inhibited melanoma B16 (33 % of mice were with complete response after 90 days) and RLS-40 tumour growth (fourfold increase in tumour doubling time) as well as metastasis.

**Conclusion:**

The anti-tumour activity of LIVP-GFP is a result of direct oncolysis of tumour cells in case of melanoma B-16 because the virus effectively replicates and destroys these cells, and virus-mediated activation of the host immune system followed by immunologically mediated destruction of of tumour cells in case of lymphosarcoma RLS-40. Thus, the recombinant vaccinia virus LIVP-GFP is able to inhibit the growth of malignant cells with the MDR phenotype and tumour metastasis when administered in the early stages of tumour development.

**Electronic supplementary material:**

The online version of this article (doi:10.1186/s12967-016-1002-x) contains supplementary material, which is available to authorized users.

## Background

The armamentarium to treat cancer today includes a variety of methods such as surgery, radio- and chemo- and targeted therapy. One of the important problems associated with anticancer therapy is the emergence of tumour cell populations that are heterogeneous and resistant to a broad spectrum of chemotherapeutics, which leads to cancer recurrence. In addition, the resistance of cancer cells to targeted therapeutics has recently been demonstrated. Several mechanisms are known to mediate tumour resistance to anticancer drugs, including increased DNA damage repair [1], reduced apoptosis [2], altered drug metabolism [3], and increased efflux of anticancer agents that enter the cells by diffusion to the plasma membrane [4]. Efflux of anticancer agents refers to multidrug resistance (MDR) that is acquired after intensive chemotherapy. One of the mechanisms of MDR is the overproduction of P-glycoprotein (P-gp), which acts as an efflux pump for various anticancer drugs [1, 5, 6]. MDR severely limits the effectiveness of chemotherapy and is responsible for the overall poor efficacy of cancer treatment [7–9]. Faced with these challenges, the development of more effective anti-cancer drugs capable of coping with MDR is an urgent task for the scientific community.

The genetic modification of oncolytic viruses (OVs) is considered to be one of the most exciting new areas of cancer therapy [10–12]. Currently, viruses from nine different families are being used for anti-cancer therapeutic development including Adenoviridae, Picornaviridae, Herpesviridae, Paramyxoviridae, Parvoviridae, Reoviridae, Poxviridae, Retroviridae and Rhabdoviridae [13–16]. The OVs have specific tissue tropisms enabling them to selectively infect and kill tumour cells with minimal impact on normal tissue [17]. Given that the mechanism of OVs not only involves direct tumour cell destruction, but also the indirect eradication of target cells expressing viral antigens as a result of immune recognition of these cells, one could assume that the utilization of OVs in MDR tumour therapy is likely to succeed.

Vaccinia virus has some very attractive advantages among other oncolytic virus candidates [18]. Above all, it should be noted that the virus has a widespread historical use due to the Smallpox Eradication Program [19, 20]. At the same time, such peculiarities of the vaccinia virus life cycle such as rapid replication cycle taking place in the cell cytoplasm, extracellular enveloped viral particles having the extra envelope formed with host complement control proteins, and large capacity of the viral DNA to tolerate the insertion or/and deletion of large genes make the virus an appealing oncolytic agent [18, 21, 22]. The efficacy of using oncolytic viruses in therapy of several cancer types has been established [23–25]. Antitumour drugs based on oncolytic viruses are currently undergoing various phases of clinical trials [26, 27]. Nevertheless, there is relatively poor information about utilizing oncolytic viruses to treat cancers displaying the MDR phenotype.

In this study, we examined the efficacy of genetically modified vaccinia virus (VACV) with disruption of the viral thymidine kinase gene (*TK*) with respect to cancer cells with the MDR phenotype in vitro and in vivo. We demonstrated that vaccinia virus administered in the early phase of tumour development is able to quite successfully suppress the growth of MDR malignant cells, as well as prevent metastasis and activate the host immune system.

## Methods

### Cell lines and virus

Mouse melanoma B-16-F10 (here and after B-16), mouse lymphosarcoma RLS, drug resistant mouse lymphosarcoma RLS-40, human cervical carcinoma KB-3-1, drug resistant human cervical carcinoma KB-8-5, African green monkey kidney fibroblast CV-1 cells were obtained from the Culture Collection of the Institute of Chemical Biology and Fundamental Medicine, Siberian Branch of the Russian Academy of Sciences. B-16, RLS and KB-3-1 cells were cultured in IMDM containing 10 % foetal calf serum (FCS) and with antibiotic–antimycotic solution (100 U/mL penicillin G, 100 U/mL streptomycin, 250 ng/mL amphotericin B) at 5 % CO_2_ and 37 °C (standard conditions). RLS-40 and KB-8-5 cells were cultured in IMDM with 40 nM Vincaleukoblastine sulfate salt (Sigma) under standard conditions.

Vaccinia virus LIVP-GFP was modified by deletion of the thymidine kinase gene and by insertion of the DNA sequence encoding the green fluorescent protein (GFP) protein. The construction of mutant vaccinia virus LIVP-GFP was described recently [28].

### Cytotoxicity assay

The viability of cells after infection with virus was measured using the 3-(4,5-dimethylthiazol-2-yl)-2,5-diphenyltetrazolium bromide (MTT) (Sigma) based assay [29]. Cells were plated in 96-well plates at the following concentrations for adherent cell cultures: 9 × 10^3^ cells per well for KB-3-1 and KB-8-5, 3 × 10^3^ for B-16 and incubated under standard conditions. When cell densities reached 80 % confluence for adherent cells and for suspension cell cultures of RLS and RLS-40 at a concentration of 1 × 10^5^ cells per well, cells were washed with PBS and incubated with the virus at 1 or 10 PFU/cell in serum-free media for 1 h. Then, the medium containing the virus was removed, and the cells were incubated in the same medium containing 2 % FCS at 37 °C for 72 h. A 5 mg/mL MTT solution was added to the cells to a concentration of 0.5 mg/mL, and the cells were incubated for 3 h under standard conditions. After removal of MTT-containing medium, the crystals of formazan were dissolved in 200 μL of DMSO, and the optical density was measured using a Multiscan RC multichannel photometer (Labsystems, Finland) at wavelengths of 570 and 620 nm. The data were presented as the percentage of viable cells, with the number of cells in the control wells incubated in the absence of the virus set at 100 %.

### Microscopic studies

Confocal fluorescence 2D imaging was performed on LSM 710 confocal laser scanning microscope (Carl Zeiss, Germany) with an objective lens (×10). The argon gas laser line of 488 nm was used to excite GFP. To acquire non-confocal transmitted light images in bright field transmitted light detector (T-PMT) was used. For illustration purposes green channel was extracted from the merged pictures with the ZEN 2011 Black edition software (Zeiss).

### Flow cytometry analysis of GFP protein expression

At 24, 48 and 72 h following viral infection, cancer cells were detached by trypsin (when applicable), fixed in a 2 % solution of formaldehyde in PBS, and GFP expression was analysed with a Cytomics FC 500 CXP flow cytometer (Beckman Coulter, United States), no less than 15,000 events/sample. The cells were considered GFP-positive if the level of their fluorescence exceeded the autofluorescence of cells in the control group by at least a factor of five. The intensity of fluorescence of individual cells was measured in relative fluorescence units (RFU) at a laser excitation wavelength of 488 nm.

### Viral proliferation assay

Standard plaque forming assay was performed to quantify viral replication following infection of different cancer lines with LIVP–GFP. The cells were infected with LIVP–GFP virus at a multiplicity of infection (MOI) of 1 or 10 PFU/cells for 1 h at 37 °C and 5 % CO_2_. At 24, 48 and 72 h after infection, tumour cells were harvested and lysates were prepared by freeze-thawing and sonication in IMDM media with 2 % FBS. The viral titres were determined by plaque forming assay using CV-1 cells as described in [30].

### Tumour development

6- to 8-week-old immunocompetent female C57Bl/6 and CBA/LacSto mice (here and after C57Bl and CBA mice, respectively) were purchased from vivarium of the State Research Center of Virology and Biotechnology “VECTOR”. Animals were kept in the vivarium of the Institute of Chemical Biology and Fundamental Medicine, SB RAS, with a natural light regimen on a standard diet for laboratory animals [GOST (State Standard) R 5025892] in compliance with the international recommendations of the European Convention for the Protection of vertebrate animals used for experimental studies (1997), as well as the rules of laboratory practice in the performance of pre-clinical studies in the Russian State Standards (R 51000.3-96 and 51000.4-96). The experimental protocols were approved by the Committee on the Ethics of Animal Experiments with the Institute of Cytology and Genetics of the Siberian Branch of the Russian Academy of Sciences.

B-16 tumour cells (1.5 × 10^5^/100 μL in IMDM) were subcutaneously (sc) injected into the right flank of 6- to 8-week-old female C57Bl mice. RLS-40 tumour cells were intramuscularly injected into the right thighs of CBA mice. The mice then received 5 × 10^7^ PFU of LIVP-GFP virus in 0.1 mL of IMDM into the site of tumour implantation immediately and 4 days after implantation. The control animals received 0.1 mL of IMDM only. Animals were observed daily for any sign of toxicity, and body weight was checked twice a week. Tumour growth was recorded in three dimensions using a digital calliper. Tumour volume was calculated as [(length × width^2^)/2] and reported in mm^3^. The median survival time is defined as the animal’s life span from the inoculation of tumour cells until death. The tumour volume doubling time (TDT) calculated using the equation TDT = (t_max_−t_0_) × ln_2_/(lnV_max_−lnV_0_), where *t*_max_−*t*_0_ is the time between measurements; *V*_0_ is the volume of the tumour at the time *t*_0_; *V*_max_ is the tumour volume at the time *t*_max_.

### Vaccinia viral titres in RLS-40 tumour

CBA mice received 5 × 10^7^ PFU of LIVP-GFP virus into tumours on day 12 after tumour implantation in the right thigh. To determine the level of viral reproduction in mice without tumour implantation the control animals received the same dose of virus in the right thigh. Then the right thigh of three virus-treated animals were excised at 1, 2, 3, 4 days post injection and frozen. Samples were then homogenized in IMDM supplemented with 2 % FBS using an ULTRA-TURRAX (IKA), and supernatants were collected by centrifugation (3500*g*, 5 min, 4 °C). Viral titres were measured by standard plaque assays on CV-1 cells.

### Expression of the virus-encoded marker gene GFP in vivo

C57Bl mice were implanted with B-16 tumour cells (1.5 × 10^5^ cells in 100 μL IMDM) into the foot pad. On day 18 after cell implantation, when the median tumour volume reached approximately 100 mm^3^, a single dose of virus (1 × 10^8^ PFU in 100 IMDM) was injected intratumourally. GFP expression within tumours was monitored each 24 h post infection using a stereo fluorescence macroimaging system (Lightools Research).

### IFN-γ ELISPOT assay

The number of LIVP-GFP-specific IFN-γ-secreting cells in mice was counted using commercial ELISPOT assay kits (BD Biosciences, USA) according to the manufacturer’s instructions. Briefly, antimouse IFN-γ monoclonal antibodies were coated on multiscreen 96-well plates at 4 °C overnight. Next, the plates were washed three times and blocked for 2 h with RPMI 1640 containing 10 % FCS at room temperature. Spleen cells were obtained after the red cells in the spleen cell suspension were lysed. Then the freshly isolated splenocytes (5 × 10^5^) were transferred to each well and LIVP-GPF was added at MOI of 0.1. Cells incubated in the absence of virus were used as a negative control and cells incubated with concanavalin A (10 µg/mL) were used as positive controls. Following incubation at 37 °C for 24 h and 5 % CO_2_, the cell suspensions were aspirated, washed four times with PBST, biotinylated detection antibody was added, and the plates were incubated for 2 h at room temperature. After four washes, streptavidin horseradish peroxidase antibody was added. Following four more washes, freshly prepared 3-amino-9-ethylcarbazole substrate solution was added for 15–30 min at room temperature in the dark to yield colored spots. Finally, the reaction was stopped by thoroughly rinsing with tap water. The plates were air-dried and the number of the spots was calculated.

### Evaluation of the pathogenic and toxic effects of LIVP–GFP virus

Each of the experimental groups contained four 7-week-old female C57Bl mice. The animals were infected intraperitoneally with the virus at doses of 10^4^, 10^6^, and 10^8^ PFU/mouse in 0.2 mL of IMDM. The mice from the control group were administered with the same volume of IMDM only. After the injection, the signs of intoxication (including general condition, weight, and depression of the central nervous system) were assessed daily for 21 days.

### Histological studies

For morphometric analysis, the livers were fixed in 10 % neutral-buffered formalin, routinely processed and embedded in paraffin. Paraffin sections (5 μm) were stained with haematoxylin and eosin, microscopically examined and scanned. Images were obtained using a microscope Axiostar plus equipped with an Axiocam MRc5 digital camera (Zeiss, Germany).

The number and area of internal metastases and destructive changes in the liver were quantified in 10–15 randomly selected microscopic fields in each specimen.

The percentages of the internal metastatic areas were determined relative to the total area of each liver section (magnification 100×) using Adobe Photoshop software. Stereological quantification of the liver samples was performed by point counting, using a closed test-system at a 400× magnification. The test-system used had 100 testing points in a testing area equal to 3.2 × 106 μm^2^. The volume densities (Vv) of normal liver parenchyma, hepatocytes with degenerate and necrotic changes and numerical density (Nv) of binuclear hepatocytes reflecting the regeneration capacity of the liver were evaluated as described in [31].

### Evaluation of cytokine levels in the blood serum of mice

Blood collection of mice was carried out under anaesthesia by a heparinised capillary pipet (No. 554/20, Assistent, Sondheim, Germany) via the retroorbital sinus vein. Blood serum was prepared by clot formation at 37 °C for 30 min and at 4 °C overnight followed by clot discard and serum centrifugation (2000*g*, 4 °C). Serum samples were stored at −20 °C until analysis. The levels of TNF-α, IL-6, GM-CSF and IFN-γ in the blood serum of mice were measured using Colorimetric ELISA Kits (ThermoScientific, USA) according to the manufacturer’s protocols. Absorbance was measured at 450 nm using a plate-reading spectrophotometer Multiscan RC multichannel photometer (Labsystems, Finland).

### Statistical analyses

A two-tailed Student’s t test was used for statistical analysis except for comparison of survival curves, where a Wilcoxon-Rank test was used. P values of ≤0.05 were considered statistically significant.

## Results

The recombinant VACV strain LIVP-GFP containing the *gfp* gene inserted in the thymidine kinase locus of the virus was constructed at the State Research Center of Virology and Biotechnology “VECTOR” [28]. The insertion of *gfp* was verified by sequence analysis as well as GFP production in the CV-1 African green monkey cell line infected with the virus. The strain was deposited in the Vector’ Collection of Cultures of Microorganisms and called LIVP–GFP. Insertion of the DNA sequence encoding GFP into the thymidine kinase (TK) gene significantly improves tracking of the virus without interfering with its ability to replicate. Moreover, insertion of the GFP gene into the TK gene of VACV significantly reduces its ability to reproduce in the majority of normal cells, because viral replication is dependent on cellular thymidine kinase, which is transiently expressed in normal cells during S phase of the cell cycle [32]. Most of the tumour cells constitutively express thymidine kinase, allowing the recombinant virus with defective thymidine kinase gene to replicate selectively in these cells [33].

### Cytotoxicity of LIVP-GFP with respect to human and mouse cancer cell lines

To determine the antitumour potential of vaccinia virus strain LIVP–GFP, we examined its cytotoxic behaviour (oncolytic activity) with respect to tumour cells of different origin: B-16 (murine melanoma), KB-3-1 (human cervical carcinoma), RLS (murine lymphosarcoma), as well as tumour cell lines with the multidrug resistance phenotype (MDR): КB-8-5 (human cervical carcinoma) [34] and RLS-40 (murine lymphosarcoma) [35]. KB-8-5 is cell line generated from the KB-3-1 cell line in the presence of 10 ng/ml colchicine and more resistant to colchicine than its parental cell line and cross-resistant to adriamycin, vincristine, vinblastine, actinomycin D, and puromycin [34]. The MDR phenotype of KB-8-5 cells is associated with overexpression of the *mdr1* gene followed by overexpression of the ATP-binding cassette (ABC) transporter P-glycoprotein (ABCB1) [36]. The MDR of the RLS-40 murine lymphosarcoma cells (RLS parental line) is also associated with overexpression of ABC-transporter genes *mdr1a/mdr1b* [37]. It should be noted that RLS cells are also drug resistant, but mainly due to the increased expression of Bcl-2 protein, which is a member of the anti-apoptotic BCL-2 family of proteins [37]. Obtained vinblastine, doxorubicin and cytarabine IC50 values were 50, 46 and 3 times higher for the RLS-40 cell line than the values in the parental line, respectively [37].

**Fig. 1:**
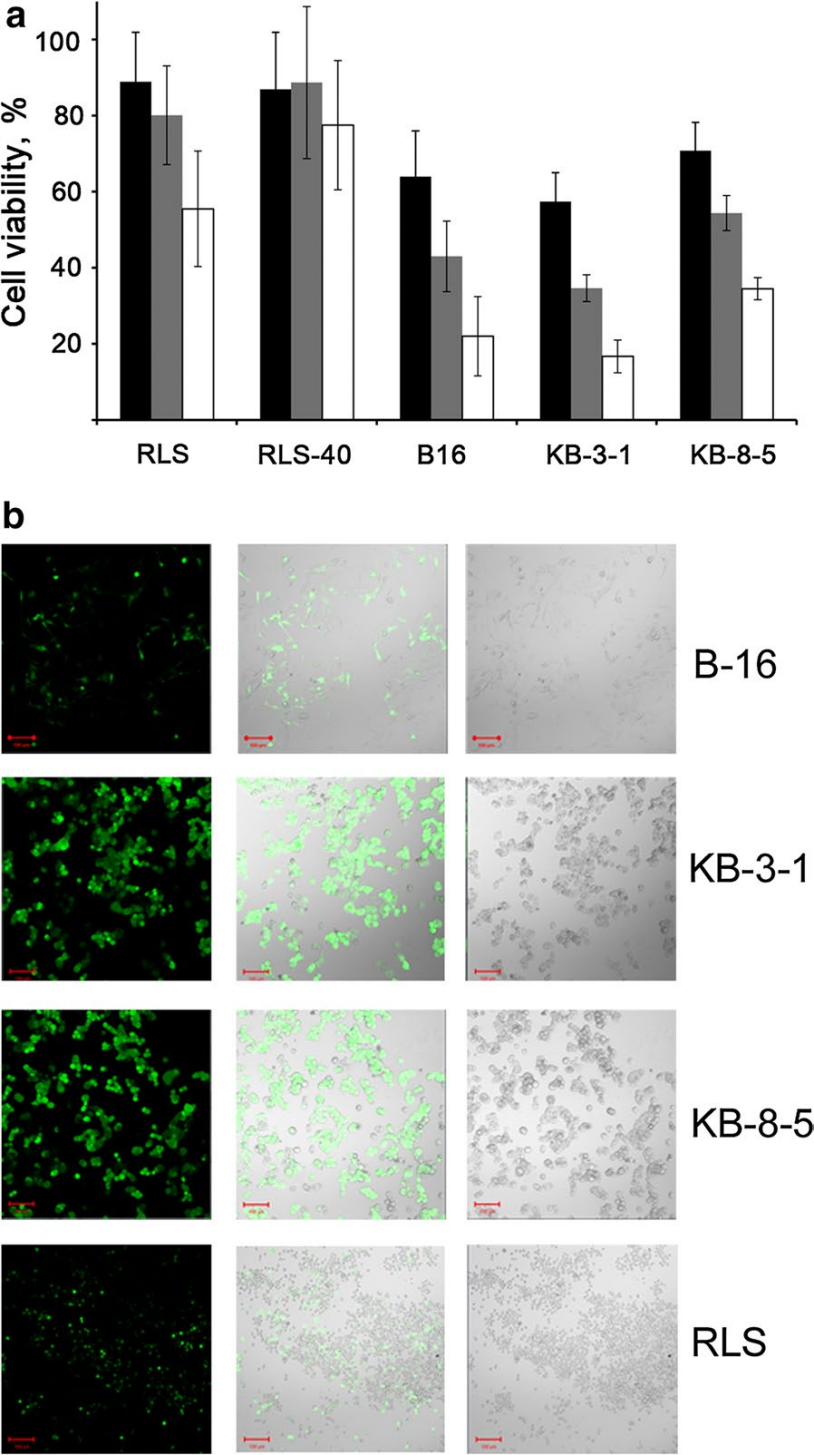
Cytopathic effect of LIVP–GFP on different tumour cells. **a** MTT assay data. The cells were infected with LIVP–GFP virus at MOI of 1. The mock-infected cells were used as a control, and the number of viable cells in the control was set at 100 %. The values are the mean ± SEM of triplicate samples. **b** Confocal fluorescent (*Left*) and non-confocal transmitted light images in bright field (*Right*) (merge is shown in the *middle*) of tumour cells 24 h post infection with LIVP-GFP at MOI of 1

The degree of tumour cell killing during the development of infection was determined 24, 48 and 72 h after the infection with the virus LIVP–GFP (MOI 1) using the MTT assay (Fig. 1). B-16 and KB-3-1 cells were the most susceptible to the virus, having only 57 and 64 % of surviving cells at 24 hpi, and 22 and 17 % at 72 hpi, respectively. The susceptibility of the MDR + KB-8-5 and RLS-40 cells was lower in comparison with the parental lines. The virus destroyed 65 % of the KB-8-5 cells by 72 hpi, whereas 83 % of the parental KB-3-1 cell died under these conditions. Both RLS (increased expression of *bcl*-*2*) and RLS-40 (overexpression of *mdr1a/mdr1b genes*) lymphosarcoma cells showing different types of drug resistance were less susceptible to the virus than KB-3-1 and B-16 cells. Moreover, approximately 50 % of RLS cells died by 72 hpi, whereas the RLS-40 cell population was reduced by 20 % at this time point. Thus, one can conclude that the cells with the MDR phenotype are less susceptible to the virus.

Confocal microscopy was used to follow the development of infection in the cells. Figure 1b displays representative images of the cells 24 hpi obtained in fluorescent and bright field modes. It can be seen that in populations of B-16, KB-3-1 and KB-8-5 but not RLS cells efficient expression of viral proteins (based on the appearance of GFP-positive cells) takes place. At this time point (24 hpi) a number of cells already changed their shape (cell blebbing) thus indicating that the cells are close to collapse. It is worth mentioning that cell blebbing is observed for cells with the brightest green fluorescence corresponding to the development of infection. The data of MTT assay coincide well with confocal microscopy data.

### Viral replication

**Fig. 2:**
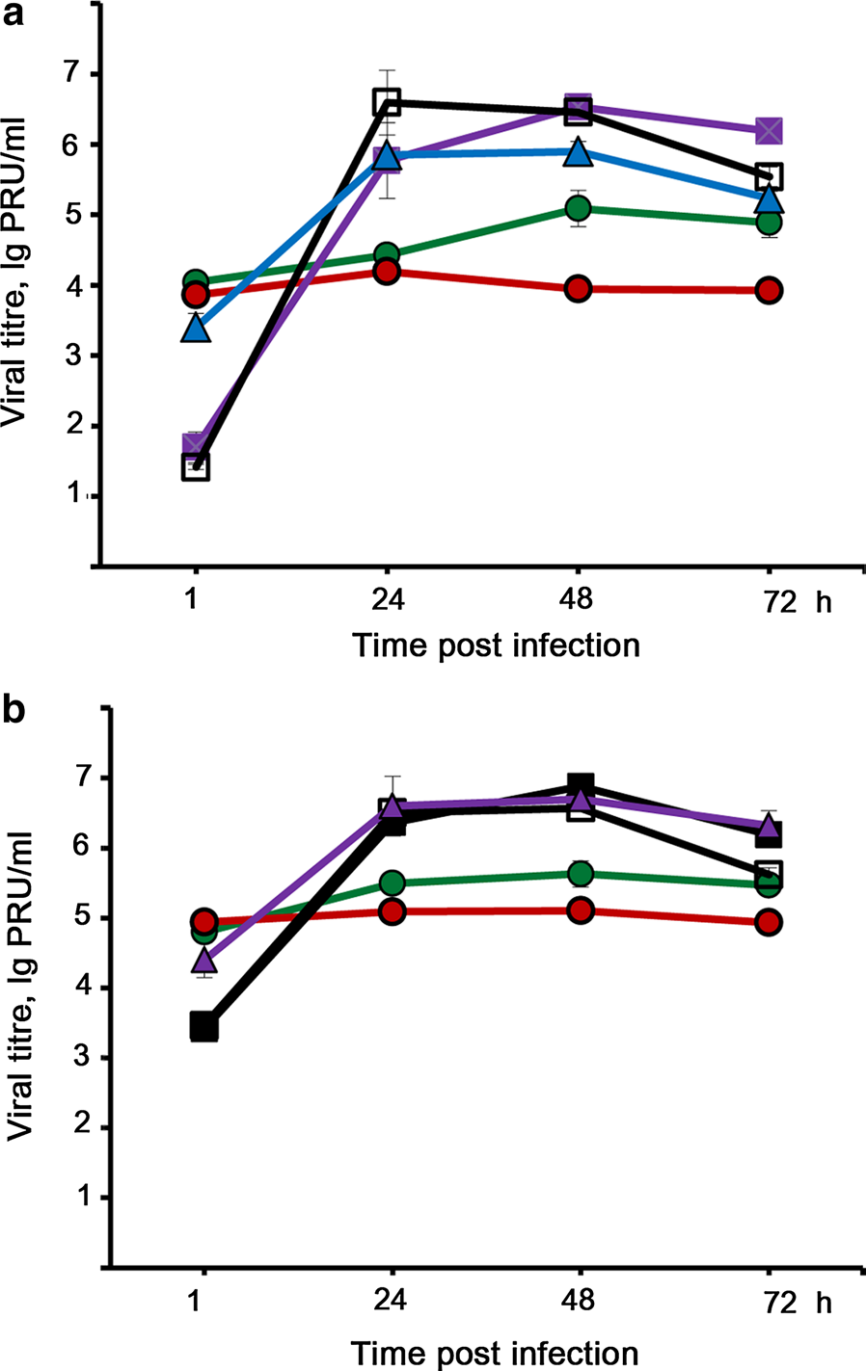
Development of LIVP-GFP infection in various tumour cells. Development of LIVP-GFP infection in RLS (*green circles*), RLS-40 (*red circles*), KB-3-1 (*violet squares*), KB-8-5 (*black line* with *open squares*) and melanoma B-16 (*blue triangles*) tumour cells at MOI of 1 (**a**) and MOI of 10 (**b**). Cells were incubated with virus for 1 h, washed with PBS and incubated up to the analysis in IMDM supplemented with 2 % FBS. Viral titre was measured by PFU assay. Data of three independent experiments are presented

The cytopathic effect of the virus depends on the efficiency of the development of productive viral infection, which can be estimated by the accumulation of viral particles in the infected cells. We compared LIVP-GFP reproduction in MDR-positive KB-8-5 and RLS-40 cells with matched parental KB-3-1 and RLS cells, as well as with B-16 murine melanoma cells. A maximum reproduction of the virus at MOI 1 was observed in KB-8-5 and KB-3-1 cells, indicating an efficient development of viral infection in these cells. The viral titre in KB-3-1 and KB-8-5 cells reached 6.1 and 6.9 lg PFU/mL, respectively (Fig. 2a). A tenfold MOI increase did not result in a concomitant increase in viral titre (Fig. 2b). The viral titre increased in B-16 cells in a similar way where values reached 5.8 lg PFU/ml during the first 24 h after infection and remained the same at 48 hpi. In turn, viral reproduction in lymphosarcomas RLS and bRLS-40 was much less effective: no effective viral production was detected in RLS-40 cells at any MOI and only an increase by an order of magnitude in viral titre was observed in infected RLS cell populations at 48 hpi and at MOI 1 (Fig. 2a).

### Analysis of expression of viral protein in infected cells

The level of susceptibility of tumour cells to infection induced by LIVP–GFP in vitro was evaluated using flow cytometry based on the appearance of GFP-positive cells (i.e., cells in which the viral proteins are expressed).

**Fig. 3:**
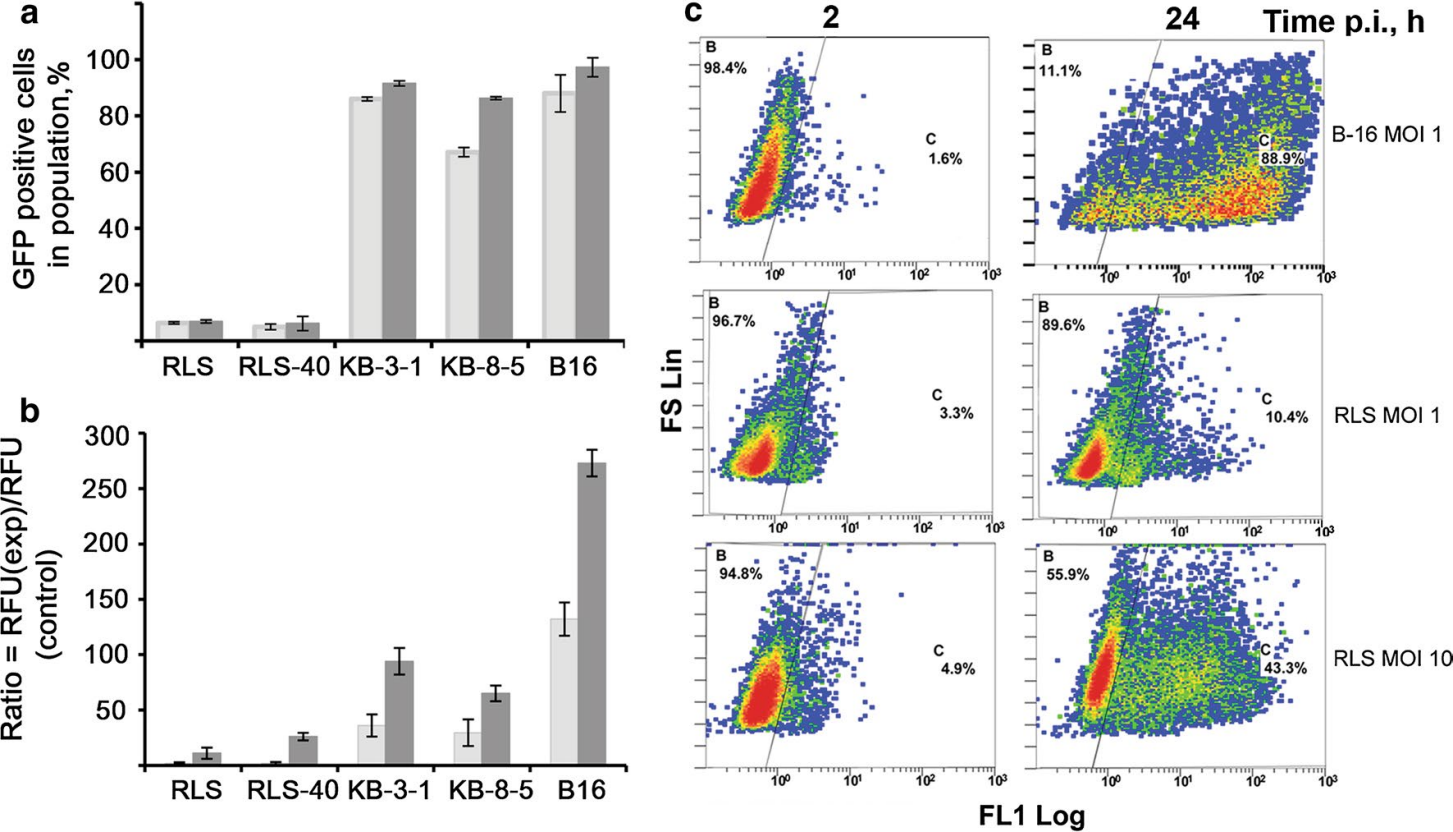
Expression of LIVP-GFP viral protein in various cell lines. The flow cytometry data. **a** Cells were infected with LIVP-GFP (MOI 1), and the percentage of GFP-positive cells was evaluated 24 h (*grey bars*) and 48 h (*dark grey bars*) post infection (hpi). GFP-positive cells were those with fluorescence intensities at least twice that of the autofluorescence intensities of non-infected cells. **b** Mean fluorescence intensities of the cell population infected with LIVP-GFP at MOI of 1 (*grey bars*) and MOI of 10 (*dark grey bars*) in comparison with the control cells at 24 hpi. **c** The original images of flow cytometry showing development of LIVP-GRF infection in tumour cells 2 and 24 hpi

A progressive increase in the number of cells expressing GFP was detected for B-16, KB-3-1 and KB-8-5 cell lines and 86 % of KB-3-1, 67 % of KB-8-5 and 89 % of B-16 cells produced GFP within 24 hpi (cells were infected at MOI 1) (Fig. 3a, c). GFP production rates were significantly less for RLS and RLS-40 cells: 7–9 % of RLS cells and 6–8 % of RLS-40 cells expressed GFP by 24 and 48 hpi. The mean fluorescence intensity (MFI) in B-16, KB-3-1 and KB-8-5 cells was much higher than the intensity in RLS/RLS-40 cells at MOI 1 (Fig. 3b, c). A tenfold MOI increase resulted in a significant increase of GFP-positive RLS and RLS-40 cells, (37 and 60 %, respectively; primary data shown in Additional file 1: Figure S1) and significantly altered the MFI for these cell lines (Fig. 3b).

### Analysis of GFP expression in vivo

**Fig. 4:**
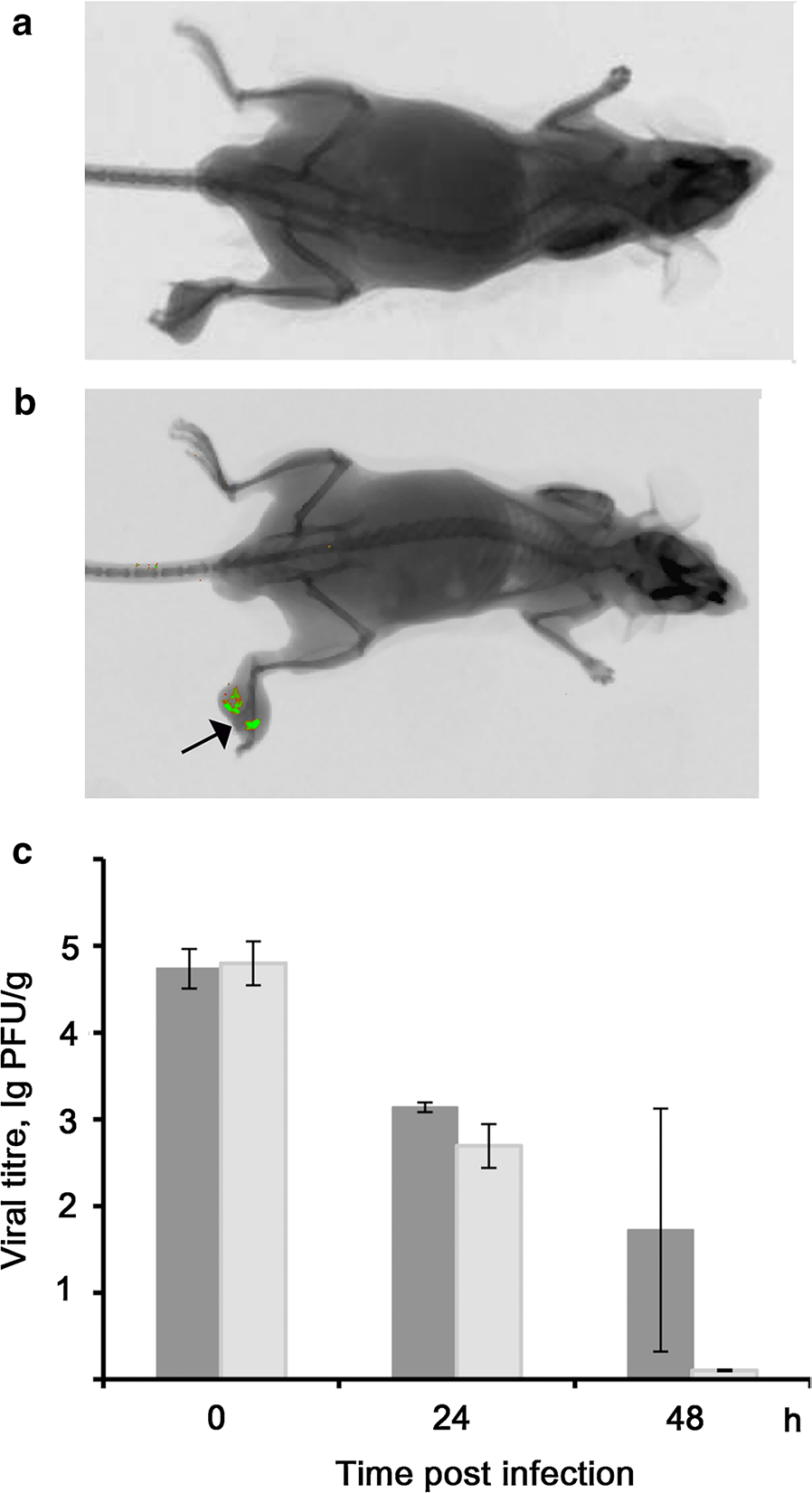
Development of LIVP-GFP virus in mice in the place of injection. **a**, **b** Imaging of animals with melanoma B-16 cells implanted in the mouse footpads followed by LIVP-GFP virus administration. Mice were imaged at 1 (**a**) and 5 (**b**) days post infection. The *arrow* shows the fluorescence of the tumour area infected with LIVP-GFP. **c** The titre of LIVP-GFP virus in the place of injection of mice with intramuscularly implanted RLS-40 cells (*dark grey*) and in healthy animals (*grey*) receiving the same dose of virus intramuscularly. The data of two independent experiments are presented

Analysis of virus replication in an immunocompetent in vivo model is an important step in the estimation of antitumour efficacy of LIVP-GFP virus. A total of 1.5 × 10^5^ of melanoma B-16 cells was implanted subcutaneously into the footpad of C57BL/6 mice. After 18 days, LIVP-GFP was injected into formed tumours. In vivo imaging was performed on days 1, 2, 3, 5 and 8 after virus injection. Expression of viral proteins monitored by GFP expression levels was observed on day 3 after intratumoural virus injection and continued well-marked up to day 5 (Fig. 4a, b). In other organs and tissues fluorescence was not detected.

Due to the relatively low sensitivity of the in vivo imaging, the dynamics of virus replication in RLS-40 tumours was analysed by determination of the virus titre in tumour tissue homogenates for 5 days after intratumoural injection of the virus (Fig. 4b). Virus injection was performed on day 12 after tumour implantation when the tumour became palpable. The data on the reproduction of the virus in vivo confirmed the results obtained in vitro. The amount of infectious virus in the samples collected at 24 and 48 hpi was continuously decreased and no virus was detected in the tumour at 72 hpi.

### LIVP-GFP therapeutic efficacy analysis

Pathogenic and toxic properties of the virus were evaluated prior to the study on its antitumour effect. Tumour-free C57Bl/6 and CBA mice were intraperitoneally injected with different doses of the virus. A single injection of the virus in a maximum dose of 10^8^ PFU/mouse did not cause significant weight loss, changes in general condition or death of the mice, indicating low toxicity of the virus.

**Fig. 5:**
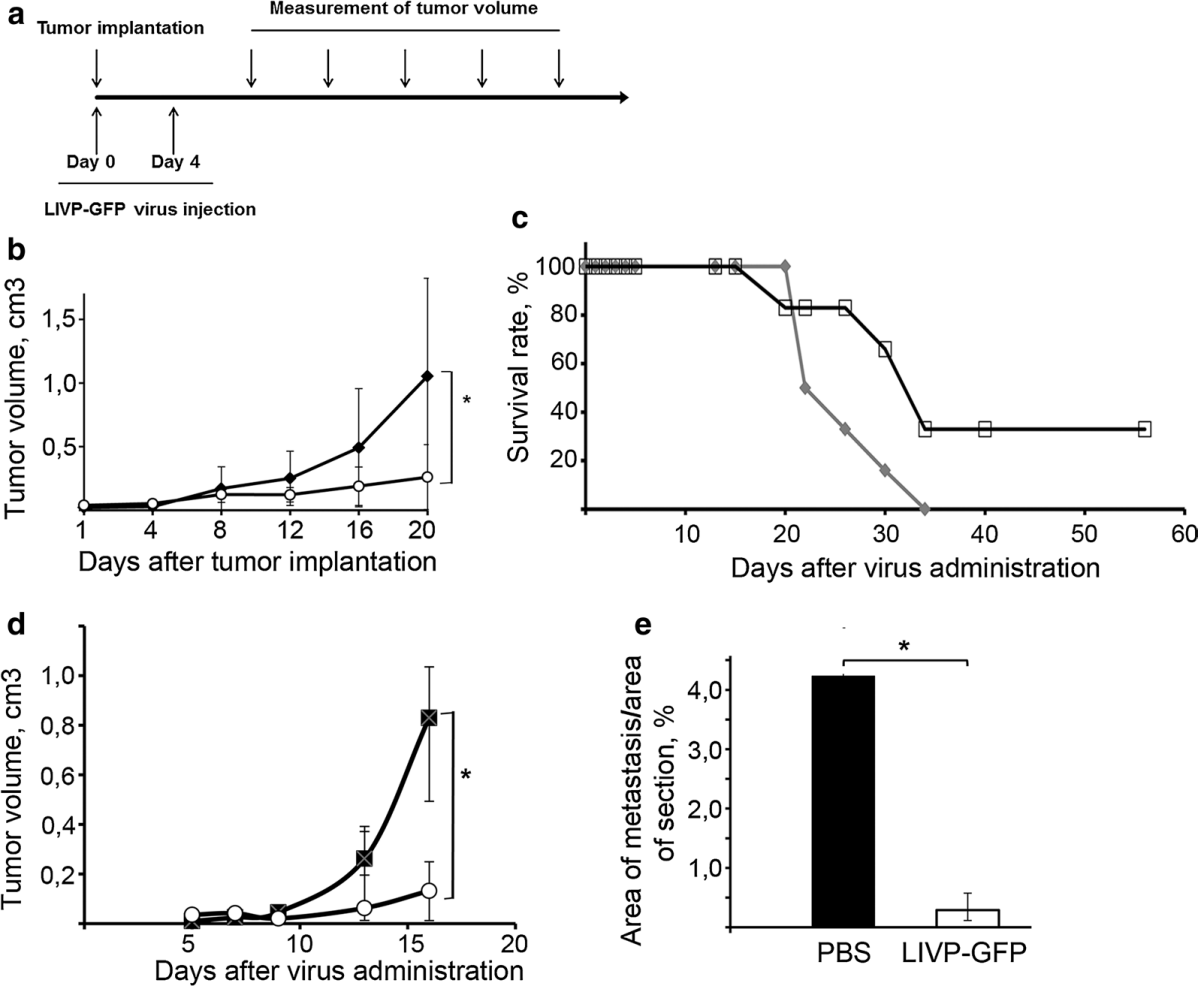
The effects of LIVP-GFP virus on tumour growth, metastasis development and lifespan of tumour-bearing mice. **a** Scheme of the experiments. **b**, **d** Inhibition of melanoma B-16 (**b**) and lymphosarcoma RLS-40 (**d**) tumour growth after treatment with LIVP-GFP. Mice with tumours implanted subcutaneously for melanoma B-16 or intramuscularly for RLS-40 received two injections of either LIVP-GFP or PBS intratumourally. Tumour volume was monitored every other day, and the mean ± SD are shown;* asterisk* denotes statistically significant difference with P < 0.05. **c** Lifespan of mice with melanoma-B-16 treated with LIVP-GFP (*open squares*) or PBS (*grey rhombus*). Mouse survival was monitored for 60 days after tumour implantation. **e** The effect of LIVP-GFP (*white bar*) on metastasis development in mice with RLS-40 tumour (*black bar* mice received PBS)

The antitumour effect of LIVP-GFP at 5 × 10^7^ PFU dose was evaluated in immunocompetent mice using two generated tumour models of B16 melanoma/C57Bl and lymphosarcoma RLS-40/CBA without and with the MDR phenotype, respectively. Melanoma B-16 tumour cells were implanted intradermally in a dose of 2 × 10^5^ cells/mouse. The virus (n = 6/group) or PBS (n = 7/group) was injected intradermally immediately after implantation and intratumourally 4 days after cell implantation (Fig. 5a). Tumour growth was quantitated by measuring tumour volume every 2–3 days. Figure 5b shows the dynamics of primary tumour growth in mice receiving LIVP-GFP or PBS. The results show that despite the obvious scatter in the data obtained by measuring tumour size, the average tumour volume was significantly smaller in the virus-treated group than in the control group (Fig. 5b). It should be noted that about half of the mice in virus-treated groups responded to the treatment, and virus therapy resulted in complete inhibition of tumour development in 33 % of the animals (Fig. 5c). Mice with complete responses were followed for up to 90 days with no evidence of tumour relapse or any toxicity attributable to the therapy.

To elucidate the antitumour activity induced by LIVP-GFP in replication-resistant lymphosarcoma RLS-40, the mice were intramuscularly implanted with RLS-40 cells and injected with the virus (n = 10/group) or PBS (n = 10/group) in the same location immediately after and 4 days after the implantation (Fig. 5a). Several mice (n = 3) were taken for histological examination on day 15 after the implantation. The data show a significant delay in tumour growth in the virus-treated animals (Fig. 5d). Analysis of tumour growth dynamics showed a 77 % reduction in tumour growth on day 13 after tumour initiation in the groups of mice treated twice with virus. At the end of the experiment, inhibition of tumour growth in this group was almost 85 %. The parameter that characterizes the intensity of tumour growth is the tumour doubling time (TDT). In the animals of the control group TDT was 1.7 ± 1.6 days, whereas in the animals from the experimental group TDT was 7.8 ± 3.9 days, indicating that antitumour therapy is efficient using LIVP-GFP in RLS-40 tumours.

**Fig. 6:**
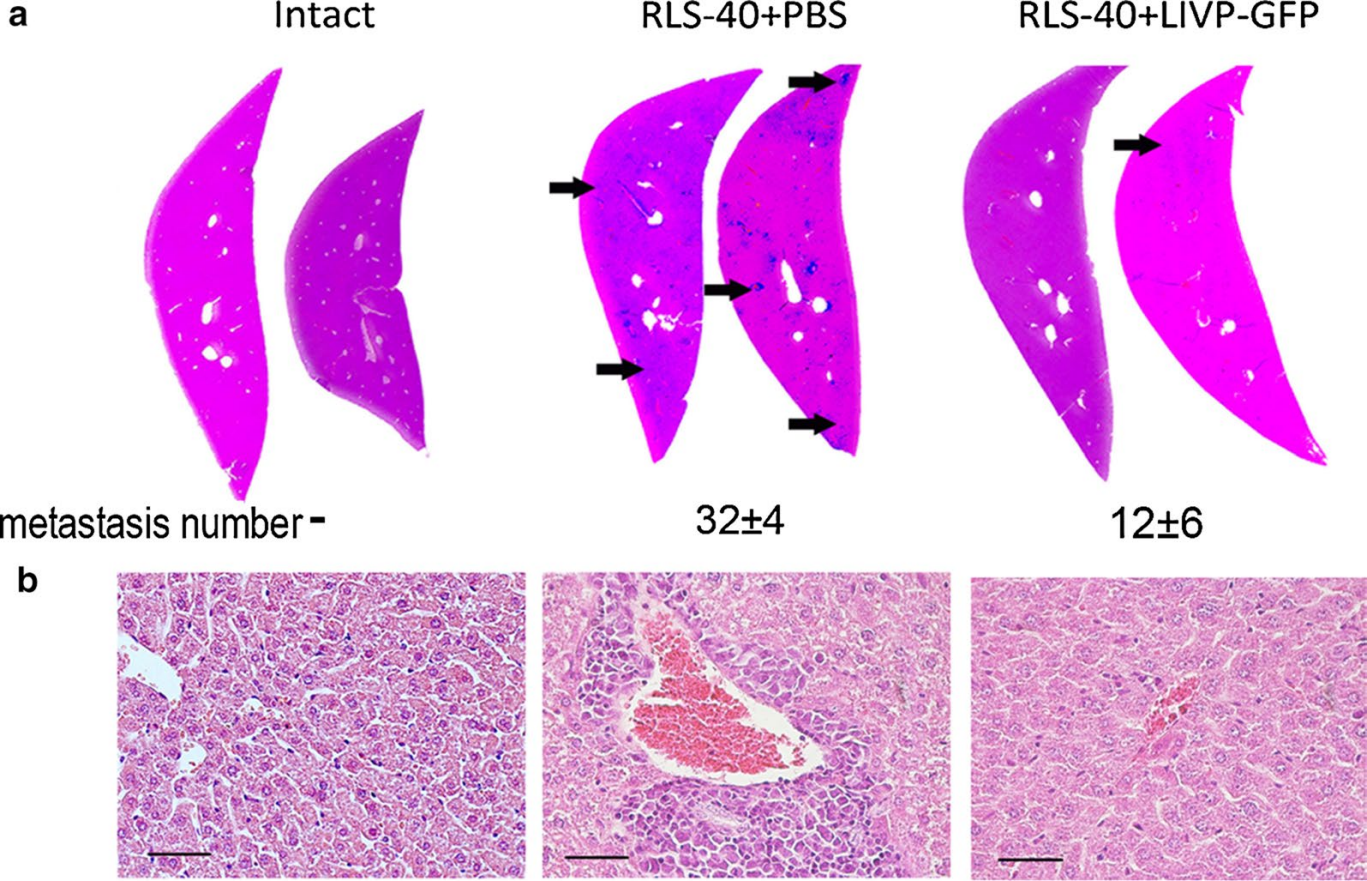
The effect of LIVP-GFP on metastasis development in liver of mice with RLS-40. **a** Liver sections of intact mice or mice with RLS-40 that received intratumoural injections of PBS or LIVP-GFP. The number of metastases found in each group of mice is shown below the corresponding section. *Arrows* show the most visible metastasis. Haematoxylin and eosin staining. **b** Histopathological analysis of the liver tissue of intact (*left*) or RLS-40-bearing mice receiving PBS (*centre*) or LIVP-GFP (*right*)

Morphometric analysis of histological sections of the livers of experimental animals revealed that the number of metastases in the group of mice treated with LIVP-GFP fluctuated significantly from 3 to 24, however the relative area occupied by metastases was almost tenfold less in this group in comparison to the PBS-treated group (Fig. 5e). Histopathological analysis of the liver tissue of RLS-40-bearing mice treated with PBS revealed diffuse and focal metastatic infiltration of the liver by tumour cells located predominantly surrounding the blood vessels (Fig. 6). Development of RLS-40 metastatic infiltration of the liver by tumour cells treated with PBS was accompanied with severe destructive changes and disturbed circulation in the adjacent liver tissue (dilatation and congestion in central vein, destruction of hepatic lobule structure, protein degeneration of hepatocytes, frequent monocellular and focal necrosis). The treatment of RLS-40-bearing animals with LIVP-GFP was accompanied by a less pronounced infiltration of the liver parenchyma by tumour cells compared to that of the control animals. Moreover, intratumoural injections of virus were associated with a decrease in the percentage and depth of dyscirculatory and destructive changes in the liver parenchyma. The portion of hepatocytes with dystrophic changes (moderate protein dystrophy) and necrosis of the liver parenchyma (rare monocellular necrosis) was reduced. The volume density of total destructive changes in the liver of the RLS-40-bearing animals treated with LIVP-GFP decreased 2.1-fold in comparison with the RLS-40-bearing mice without treatment (see Additional file 1: Table S1).

### Evaluation of LIVP-GFP-associated immune response activation

**Fig. 7:**
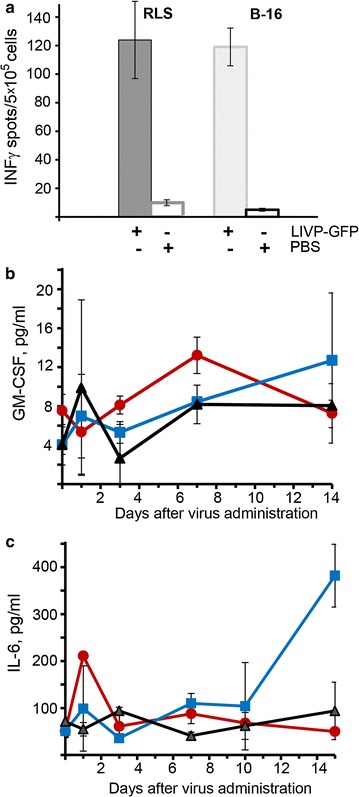
Effect of LIVP-GFP treatment on the immune responses of tumour-bearing mice. **a** Summary data showing a significant increase of the number of IFN-γ-ransecreting splenocytes in LIVP-GFP-treated mice (n = 6) with RLS-40 or with melanoma B-16 tumours. Comparison of immune-related proteins GMC-SF (**b**) and IL-6 (**c**) in the blood serum of RLS-40 bearing mice during the treatment with LIVP-GFP (the experimental scheme was shown in Fig. 5a):* red circles* and* blue squares* for RLS-40 bearing mice treated with LIVP-GFP and PBS, respectively;* black triangles*—healthy mice receiving PBS. The levels of cytokines in the blood serum were measured by ELISA. For each day, the value of MEAN ± SEM is shown

Taking into account the fact that the virus effectively inhibits the development of tumours in mice implanted with lymphosarcoma RLS-40 cells, but does not effectively replicate in these cells (Figs. 3, 4b), in contrast to the productive viral replication that occurs in melanoma B-16 cells, one could assume that the host immune system might be a factor determining the efficacy of the virotherapy. To test this hypothesis, mice were implanted with lymphosarcoma RLS-40 cells into the right femoral muscle or subcutaneously with melanoma B-16 cells. The virus was injected in a dose of 5 × 10^7^ PFU/mouse in the tumour site immediately after and 4 days following the implantation. The control group was injected with PBS. Splenocytes were obtained from all groups 3 days after the second virus injection, and the activation of the T-cell host immune system was evaluated by quantifying the number of virus-specific IFN-γ-secreting cells (Fig. 7a). LIVP-GFP injection resulted in a ten-fold increase of virus-specific IFN-γ-secreting cells in both B-16 and RLS-40 tumour-bearing mice, thus, indicating the stimulation of a T-cell immune response (Fig. 7a).

For further analysis of the immunological mechanisms involved in the growth inhibition of lymphosarcoma RLS-40 after virus injection, and given the evidence of the virus-mediated induction of various pro-inflammatory mediators and cytokines, we assessed the kinetics of GM-CSF, IFN-γ, TNF-α and IL-6 in the serum of RLS-40-bearing mice treated with the virus or PBS. The levels of GM-CSF, IFN-γ and TNF-α in both experimental and control groups showed no significant change during the monitoring period (Fig. 7b, data referred to GM-CSF). A significant increase in the IL-6 concentration in mice treated with PBS (but not with the virus) was observed. The elevated IL-6 levels correlated with intensive tumour growth in this group (Figs. 5d, 7c).

## Discussion

The aim of the study was to investigate LIVP-GFP replication efficacy in MDR cancer cells, its ability to destroy these cells in vitro and determine whether such virotherapy can be used to treat MDR tumours in vivo. The human carcinoma KB-8-5 cell line with *mdr1* overexpression (parental cell line KB-3-1) [34] and murine lymphosarcoma RLS-40 cell line with *mdr1a/mdr1b* overexpression [37] were chosen as a model system. The virus replication efficacy in these two cell lines was radically different. The number of GFP-producing cells corresponding to viral protein production as well as the reproduction of infectious virus in KB-3-1 and KB-8-5 cells was much higher than the same infection parameters in RLS and RLS-40 cells. However, the replication efficacy was the same in KB-3-1 and KB-8-5 cells, as was the efficacy of viral protein expression and cells killed by virus. On the basis of these results, it is likely that the virotherapy with LIVP-GFP mice bearing KB-8-5 tumours with the MDR phenotype as well as the parental tumours KB-3-1 could be successful and it remains to be tested in future experiments.

LIVP-GFP production in RLS cells increased very slightly over the observation period and reached its highest level at 48 hpi. The viral titre in RLS-40 lymphosarcoma cells remained unchanged throughout the entire period of observation. It is interesting to note that a tenfold MOI increase did not result in increased virus reproduction but only in a higher number of GFP-producing cells and MFI of infected cells. Virus replication analysis in B-16 murine melanoma cells showed relatively high susceptibility of these cells to the virus, where a MOI increase correlated with an increase in the number of newly synthesized viral particles peaking at 48 hpi (Fig. 3). Reduced efficiency of viral replication in B-16 cells as compared to KB-3-1 and KB-8-5 cells may be associated with generally less effective viral replication in murine cells than in human cells [38, 39]. In turn, the reduced susceptibility of RLS and RLS-40 shown in this study is consistent with the findings of other researchers showing the low viral replication efficacy in primary human leukocyte subsets including monocytes, B cells, NK cells (both in resting and activated states), neutrophils, and resting T cells [40–42]. Taken together, the results of in vitro analysis indicate that different tumour cell lines with MDR phenotypes demonstrated different susceptibilities to LIVP-GFP, which depend on the ability of virus to replicate in these cells.

Oncolytic vectors are supposed to destroy tumour cells in two phases: after viral replication in cells and after induction of an immune response against viral antigens expressed in tumour cells (immunologically mediated destruction). Recently, it was shown that vaccinia virus infection induces necrotic death in ovarian cancer cells [43]. Destruction of vaccinia virus infected tumor cells leads to the release of tumour associated antigens and danger signals into extra-cellular surrounding and tumour microenvironment [44]. Such danger signals could be sensed by cells of innate immune system, so it could be suggested that activation of innate immune system might take part in tumor regression in cooperation with the viral oncolytic process [45]. Moreover, most recently, Zonov et al. demonstrated that direct cell destruction have been implicated in vaccinia infection of human carcinoma A431 cells, but in Ehrlich carcinoma cells which poorly supported vaccinia virus replication the virus induced suppression of mitoses [46]. However, nowadays the mechanisms mediating death of vaccinia virus infected cells are not clear entirely. In particular, for some cancer cells it was shown that apoptosis is a main pathway of vaccinia virus infected cell death [47].

Since viral replication has not been observed in RLS-40 lymphosarcoma cells (in vitro and in vivo), the inhibition of tumour growth in immunocompetent mouse model CBA—RLS-40 lymphosarcoma after treatment with LIVP-GFP would mainly depend on the reaction of the host immune system to the virus injection. Given the high immunostimulatory activity of the virus, it was interesting to assess the effectiveness of virotherapy in such a system. A similar study was conducted by Prestwich and colleagues [48] with oncolytic reovirus and reovirus-nonpermissive tumours, which allowed the authors to focus on the role of the virus in the activation of the immune system. In addition, we compared the antitumour efficacy of LIVP-GFP in RLS-40 lymphosarcoma cells with melanoma B-16 in immunocompetent mice, taking into consideration the differential ability of LIVP-GFP to replicate and kill cells in this tumour cell line. We had previously determined that intratumoural administration of the LIVP-GFP virus resulted in a significant inhibition of tumour growth in mice bearing melanoma B-16 [28]. Inhibition of the growth of such a quick-growing tumours as melanoma B16 after a single administration of the LIVP–GFP virus indicates its high antitumour potential. Since complete tumour regression after a single administration of virus was not obtained, we optimized the viral application procedure. The virus was now injected twice: immediately after tumour implantation and 4 days after.

Usually, intravenous or intratumoural routs of vaccinia virus administration are used. Oncolytic vaccinia virus injected intravenously is able to infect and replicate within cancer cells but intravenous delivery of the virus would result in a drastic loss of the virus in the blood stream mainly due to the rapid clearance by immune system [49]. Direct intratumoural vaccinia application may be useful in treating locally aggressive tumors and now randomized dose-finding trial of Pexa-Vec in patients with advanced HCC showed promising results. In this trial Pexa-Vec was delivered by intratumoural injection [50]. Intratumoural delivery also allows using of lower virus doses and probably postpones antiviral immune response. Besides, intratumoural virus administration could provide higher multiplicity of infection of tumour cells. For these reason we used in our study direct intratumoural administration of LIVP-GFP.

Consistent with our previous results, LIVP-GFP significantly inhibited the growth of melanoma B-16 in mice. The optimization of the treatment strategy has led to an increased survival benefit: 33 % of animals in the group bearing melanoma B-16 treated with the virus showed a complete response and survived, while control animals did not survive. We also demonstrated that the administration of LIVP-GFP twice in the early stages of tumour development is able to inhibit the growth of malignant RLS-40 cells with the MDR phenotype, as well as tumour metastasis. We hypothesize that two mechanisms underlie the effects of virotherapy of these tumours. A direct oncolysis is induced by the virus in the case of melanoma B-16 because the virus effectively replicates and destroys these tumour cells, and virus-mediated activation of the host immune system is followed by immunologically mediated destruction of lymphosarcoma RLS-40. In order to exam whether the mechanism of inhibition of RLS-40 lymphosarcoma displaying the MDR phenotype was mediated by activation of the immune system, we estimated the generation CD8 T-cells after treatment of mice with LIVP-GFP.

It is known that the vaccinia virus is capable of generating a highly specific T-cell immune response [51]. According to recent studies [52], it is also known that activation of a T-cell immune response significantly contributes to the development of antitumour immunity. Therefore, it is important to evaluate the efficiency of T-cell immune response induction by the LIVP-GFP virotherapy. We observed a significant (tenfold) increase in the number of virus-specific IFN-γ -producing cells after virus injection. Although tumour-specific IFN-γ-secreting cells were not observed, it has been shown that adaptive cytotoxic T-cell responses can promote powerful activation of innate immune effector cells [53] and generate a highly immunogenic environment [39]. We assume that the stimulation of T-cell immunity resulted in a decrease in the number of metastases in animals with implanted RLS-40 cells after two injections of the virus. A reduction in the number of metastases after a CD8+ T lymphocyte increase has also been shown in Wang et al. [39]. These authors showed that removal of CD4 lymphocytes, neutrophils and NK cells populations had no effect on the anti-metastatic activity of the virus. It worth noting that results obtained with a mathematical model of virotherapy indicated that while viral oncolysis is fundamental in reducing the tumour burden, increased stimulation of cytotoxic T cells leads to a short-term reduction in tumour size, but a faster relapse [54]. It was hypothesized that viral destruction of melanoma B-16 cells resulted in more effective tumour elimination compared to the outcome of viral therapy in RLS-40 lymphosarcoma-bearing mice.

In addition to virus-specific T-cell activation, we assessed the humoral immune response in RLS-40 lymphosarcoma-bearing mice after treatment with either LIVP-GFP or PBS. The activation of the humoral immune system during virotherapy has been tested in various studies [55–57], but in general the authors analysed the levels and the spectrum of cytokines present in tumour tissue or by culturing infected tumour cells. The experiments revealed no significant fluctuations in the level of GM-CSF, IFN-γ, TNF-α during the entire period of observation; however, we found a dramatic increase in the concentration of IL-6 in mice in the control group (treatment with PBS), which coincides with a sharp increase in the size of the tumours. In contrast, IL-6 levels were the same in the experimental group as those of intact animals. IL-6 is a pleiotropic cytokine that induces an active phase of the immune response, stimulates activation of B and T lymphocytes, and regulates growth, differentiation and death of certain cell populations [58]. Binding of IL-6 to its receptor complex leads to JAK activation followed by subsequent phosphorylation and dimerization of NF-kB and STAT-3. IL-6 is one of the key cytokines involved in the proliferation and differentiation of tumour cells [59]. Moreover, it was shown that IL-6 promotes tumor growth by VEGF-dependent angiogenesis in particular via a STAT3 pathway [59–61]. Taking in account that IL-6 takes part in regulation of VEGF expression we can speculate that decreasing of level IL-6 after therapy of tumor with LIVP-GFP might lead to decreasing of VEFR’s level thus affecting tumour vasculature. Significant reducing of VEGF levels in the tumor of mice treated intravenously with vaccinia virus was shown recently [62].

IL-6 stimulates the development of many tumours, including glioma [63], multiple myeloma [64] and colorectal carcinoma [65]. IL-6 overexpression and its receptors may be associated with increased proliferation of breast cancer cells [66]. Given the role of IL-6 in the activation of androgen receptors, neuroendocrine differentiation and angiogenesis in prostate cancer development, attempts have been made to inhibit the cytokine signalling pathways using monoclonal antibodies [67]. Inhibition of tumour growth in IL-6 knockout mice also indicates the role of IL-6 in stimulation of tumour development [68]. Whether the inhibition of IL-6 is a key factor in inhibition of RLS-40 lymphosarcoma growth remains to be seen in future experiments.

An increase in the immunostimulatory properties of VACV is implemented in a number of new approaches to virotherapy use in cancer treatment. In addition to cytokines and immunostimulatory molecules embedded in the viral genome, a method of using so-called T cell-engagers that can bind T cells to tumour cells and additionally induce immune-mediated tumour cell destruction was proposed [69]. In other studies, cytokine-induced killer (CIK) cells were used as a vector for VACV systemic delivery [38, 70]. A few other approaches that can also have a direct impact on the immune responses induced by the viral vectors have been devised [71]. Furthermore, to enhance the antitumour effect of vaccinia virus Teigler with colleagues [72] suggest considering the variability of the immune response induced by the vector. Given the diversity and the effectiveness of pro-inflammatory cytokine and chemokine responses, researchers have the flexibility to choose a vector for activation of cytokines responsible for tumour growth inhibition, which in combination with other methods of virotherapy may be an effective way to eliminate cancer cells.

Understanding the mechanisms of resistance and susceptibility of various tumours to the action of oncolytic viruses, the choice of a vector for cautious activation of an immune response and the use of combination therapy to enhance the effectiveness of the virotherapy is necessary to develop such an approach to anticancer therapy and remains a subject of further research.

## Conclusions

Chemotherapy is likely to be the main treatment approach for cancer for a long time to come. An effective co-treatment in order to achieve solid regression of the tumour would be adjuvants with various mechanisms of action, and oncolytic viruses have many advantages among other candidates. The data we obtained show that the anti-tumour activity of LIVP-GFP detected in case of melanoma B-16 was a result of direct oncolysis of tumour cells because the virus effectively replicates and destroys these cells. We demonstrated that virus-mediated activation of the host immune system is followed by immunologically mediated destruction of lymphosarcoma RLS-40. Thus, the recombinant vaccinia virus LIVP-GFP is able to inhibit the growth of malignant cells with the MDR phenotype and tumour metastasis when administered in the early stages of tumour development either by direct oncolysis of tumour cells or by activation of the host immune system. In addition, the data on the immunostimulatory activity of vaccinia virus can be applied to develop more efficient schemes for virotherapy in cancer diseases.

## Additional file

10.1186/s12967-016-1002-x The original flow cytometry images showing development of LIVP-GRF infection at MOI = 1 or MOI = 10 in different tumour cells 2, 24 and 48 hpi. **Table S1.** The effect of LIVP-GFP treatment on the liver of RLS-40-bearing mice.

## References

[1] Bartkova J, Horejsi Z, Koed K, Kramer A, Tort F, Zieger K (2005). DNA damage response as a candidate anti-cancer barrier in early human tumorigenesis. Nature.

[2] Tsuruo T, Naito M, Tomida A, Fujita N, Mashima T, Sakamoto H (2003). Molecular targeting therapy of cancer: drug resistance, apoptosis and survival signal. Cancer Sci.

[3] Marin JJ, Romero MR, Martinez-Becerra P, Herraez E, Briz O (2009). Overview of the molecular bases of resistance to chemotherapy in liver and gastrointestinal tumours. Curr Mol Med.

[4] Nobili S, Landini I, Mazzei T, Mini E (2012). Overcoming tumor multidrug resistance using drugs able to evade P-glycoprotein or to exploit its expression. Med Res Rev.

[5] Marin JG, Monte MJ, Blazquez AG, Macias IR, Serrano MA, Briz O (2014). The role of reduced intracellular concentrations of active drugs in the lack of response to anticancer chemotherapy. Acta Pharmacol Sin.

[6] Pérez-Tomás R (2006). Multidrug resistance: retrospect and prospects in anti-cancer drug treatment. Curr Med Chem.

[7] Kuo MT (2007). Roles of multidrug resistance genes in breast cancer chemoresistance. Adv Exp Med Biol.

[8] Valera ET, Scrideli CA, Queiroz RG, Mori BM, Tone LG (2004). Multiple drug resistance protein (MDR-1), multidrug resistance-related protein (MRP) and lung resistance protein (LRP) gene expression in childhood acute lymphoblastic leukemia. Sao Paulo Med J.

[9] Lu C, Shervington A (2008). Chemoresistance in gliomas. Mol Cell Biochem.

[10] Albelda SM, Thorne SH (2014). Giving oncolytic vaccinia virus more BITE. Mol Ther.

[11] Buijs PR, Verhagen JH, van Eijck CH, van den Hoogen BG (2015). Oncolytic viruses: from bench to bedside with a focus on safety. Hum Vaccin Immunother.

[12] Coffin RS (2015). From virotherapy to oncolytic immunotherapy: where are we now?. Curr Opin Virol.

[13] Miest TS, Cattaneo R (2014). New viruses for cancer therapy: meeting clinical needs. Nat Rev Microbiol.

[14] Thirukkumaran C, Morris DG (2015). Oncolytic viral therapy using reovirus. Methods Mol Biol.

[15] Verheije MH, Rottier PJM (2012). Retargeting of viruses to generate oncolytic agents. Adv Virol.

[16] Chiocca EA, Rabkin SD (2014). Oncolytic viruses and their application to cancer immunotherapy. Cancer Immunol Res.

[17] Zeyaullah M, Patro M, Ahmad I, Ibraheem K, Sultan P, Nehal M (2012). Oncolytic viruses in the treatment of cancer: a review of current strategies. Pathol Oncol Res.

[18] Kim M (2015). Replicating poxviruses for human cancer therapy. J Microbiol.

[19] World Health Organization (1980). The global eradication of smallpox: final report of the global commission for the certification of smallpox eradication.

[20] Singh RK, Balamurugan V, Bhanuprakash V, Venkatesan G, Hosamani M (2012). Emergence and reemergence of vaccinia-like viruses: global scenario and perspectives. Indian J Virol.

[21] Chan WM, McFadden G (2014). Oncolytic poxviruses. Annu Rev Virol.

[22] Smith GL, Moss B (1983). Infectious poxvirus vectors have capacity for at least 25 000 base pairs of foreign DNA. Gene.

[23] Donnelly OG, Errington-Mais F, Prestwich R, Harrington K, Pandha H, Vile R (2012). Recent clinical experience with oncolytic viruses. Curr Pfarm Biotechnol.

[24] Park SH, Breitbach CJ, Lee J, Park JO, Lim HY, Kang WK (2015). Phase 1b trial of biweekly intravenous pexa-vec (jx-594), an oncolytic and immunotherapeutic vaccinia virus in colorectal cancer. Mol Ther.

[25] Gholami S, Chen CH, Lou E, Belin LJ, Fujisawa S, Longo VA (2014). Vaccinia virus GLV-1h153 in combination with 131I shows increased efficiency in treating triple-negative breast cancer. FASEB J.

[26] Russell SJ, Peng KW, Bell JC (2012). Oncolytic virotherapy. Nat Biotechnol.

[27] Pol J, Bloy N, Obrist F, Eggermont A, Galon J, Cremer I (2014). Trial watch: oncolytic viruses for cancer therapy. Oncoimmunology.

[28] Petrov IS, Goncharova EP, Kolosova IV, Pozdnyakov SG, Shchelkunov SN, Zenkova MA (2013). Antitumor effect of the LIVP-GFP recombinant vaccinia virus. Dokl Biol Sci.

[29] Park JG, Kramer BS, Steinberg SM, Carmichael J, Collins JM, Minna JD (1987). Chemosensitivity testing of human colorectal carcinoma cell lines using atetrazolium-based colorimetrie assay. Cancer Res.

[30] Frentzen A, Yu YA, Chen N, Zhang Q, Weibel S, Raab V (2009). Anti-VEGF single-chain antibody GLAF-1 encoded by oncolytic vaccinia virus significantly enhances antitumor therapy. PNAS.

[31] Sen’kova AV, Mironova NL, Patutina OA, Ageeva TA, Zenkova MA (2012). The toxic effects of polychemotherapy onto the liver are accelerated by the upregulated MDR of lymphosarcoma. ISRN Oncol.

[32] Sherley JL, Kelly TJ (1988). Regulation of human thymidine kinase during the cell cycle. J Biol Chem.

[33] Guse K, Cerullo V, Hemminki A (2011). Oncolytic vaccinia virus for the treatment of cancer. Expert Opin Biol Ther.

[34] Akiyama S, Fojo A, Hanover JA, Pastan I, Gottesman MM (1985). Isolation and genetic characterization of human KB cell lines resistant to multiple drugs. Somat Cell Mol Gene.

[35] Kaledin VI, Nikolin VP, Ageeva TA, Timofeeva OA, Filipenko ML, Ronichevskaia GM (2000). Cyclophosphamide-induced apoptosis of murine lymphosarcoma cells in vivo. Vopr Onkol.

[36] Kanzaki A, Takebayashi Y, Ren XQ, Miyashita H, Mori S, Akiyama S (2002). Overcoming multidrug drug resistance in p-glycoprotein/MDR1-overexpressing cell lines by ecteinascidin 743. Mol Cancer Ther.

[37] Mironova N, Shklyaeva O, Andreeva E, Popova N, Kaledin V, Nikolin V (2006). animal model of drug-resistant tumor progression. Ann NY Acad Sci.

[38] Thorne SH, Negrin RS, Contag CH (2006). Synergistic antitumor effects of immune cell-viral biotherapy. Science.

[39] Wang LC, Lynn RC, Cheng G, Alexander E, Kapoor V, Moon EK (2012). Treating tumors with a vaccinia virus expressing IFNβ illustrates the complex relationships between oncolytic ability and immunogenicity. Mol Ther.

[40] Byrd D, Amet T, Hu N, Lan J, Hu S, Yu Q (2013). Primary human leukocyte subsets differentially express vaccinia virus receptors enriched in lipid rafts. J Virol.

[41] Chahroudi A, Chavan R, Kozyr N, Waller EK, Silvestri G, Feinberg MB (2005). Vaccinia virus tropism for primary hematolymphoid cells is determined by restricted expression of a unique virus receptor. J Virol.

[42] Yu Q, Jones B, Hu N, Chang H, Ahmad S, Liu J (2006). Comparative analysis of tropism between canarypox (ALVAC) and vaccinia viruses reveals a more restricted and preferential tropism of ALVAC for human cells of the monocytic lineage. Vaccine.

[43] Whilding LM, Archibald KM, Kulbe H, Balkwill FR, Öberg D, McNeish IA (2013). Vaccinia virus induces programmed necrosis in ovarian cancer cells. Mol Ther.

[44] Al Yaghchi C, Zhang Z, Alusi G, Lemoine NR, Wang Y (2015). Vaccinia virus, a promising new therapeutic agent for pancreatic cancer. Immunotherapy.

[45] Worschech A, Haddad D, Stroncek DF, Wang E, Marincola FM, Szalay AA (2009). The immunologic aspects of poxvirus oncolytic therapy. Cancer Immunol Immunother.

[46] Zonov E, Kochneva G, Yunusova A, Grazhdantseva A, Richter V, Ryabchikova E (2016). Features of the antitumor effect of vaccinia virus lister strain. Viruses.

[47] Greiner S, Humrich JY, Thuman P, Sauter B, Schuler G, Jenne L (2006). The highly attenuated vaccinia virus strain modified virus Ankara induces apoptosis in melanoma cells and allows bystander dendritic cells to generate a potent anti-tumoral immunity. Clin Exp Immunol.

[48] Prestwich RJ, Ilett EJ, Errington F, Diaz RM, Steele LP, Kottke T (2009). Immune-mediated antitumor activity of reovirus is required for therapy and is independent of direct viral oncolysis and replication. Clin Cancer Res.

[49] Ferguson MS, Lemoine NR, Wang Y (2012). Systemic delivery of oncolytic viruses: hopes and hurdles. Adv Virol.

[50] Breitbach CJ, Moon A, Burke J, Hwang TH, Kirn DH (2015). A phase 2, open-label, randomized study of Pexa-Vec (JX-594) administered by intratumoral injection in patients with unresectable primary hepatocellular carcinoma. Methods Mol Biol.

[51] Xu R, Johnson AJ, Liggitt D, Bevan MJ (2004). Cellular and humoral immunity against vaccinia virus infection of mice. J Immunol.

[52] Yong X, Xiao YF, Luo G, He B, Lü MH, Hu CJ (2012). Strategies for enhancing vaccine-induced CTL antitumor immune responses. J Biomed Biotechnol.

[53] Shanker A, Verdeil G, Buferne M, Inderberg-Suso EM, Puthier D, Joly F (2007). CD8 T cell help for innate antitumor immunity. J Immunol.

[54] Kim PS, Crivelli JJ, Choi IK, Yun CO, Wares JR (2015). Quantitative impact of immunomodulation versus oncolysis with cytokine-expressing virus therapeutics. Math Biosci Eng.

[55] Ehrig K, Kilinc MO, Chen NG, Stritzker J, Buckel L, Zhang Q (2013). Growth inhibition of different human colorectal cancer xenografts after a single intravenous injection of oncolytic vaccinia virus GLV-1h68. J Transl Med.

[56] Guillerme JB, Boisgerault N, Roulois D, Ménager J, Combredet C, Tangy F (2013). Measles virus vaccine-infected tumor cells induce tumor antigen cross-presentation by human plasmacytoid dendritic cells. Clin Cancer Res.

[57] Li J, O’Malley M, Urban J, Sampath P, Guo ZS, Kalinski P (2011). Chemokine expression from oncolytic vaccinia virus enhances vaccine therapies of cancer. Mol Ther.

[58] Trikha M, Corringham R, Klein B, Rossi JF (2003). Targeted anti-interleukin-6 monoclonal antibody therapy for cancer: a review of the rationale and clinical evidence. Clin Cancer Res.

[59] Guo Y, Xu F, Lu T, Duan Z, Zhang Z (2012). Interleukin-6 signaling pathway in targeted therapy for cancer. Cancer Treat Rev.

[60] Angelo LS, Kurzrock R (2007). Vascular endothelial growth factor and its relationship to inflammatory mediators. Clin Cancer Res.

[61] Saidi A, Hagedorn M, Allain N, Verpelli C, Sala C, Bello L (2009). Combined targeting of interleukin-6 and vascular endothelial growth factor potently inhibits glioma growth and invasiveness. Int J Cancer.

[62] Hou W, Chen H, Rojas J, Sampath P, Thorne SH (2014). Oncolytic vaccinia virus demonstrates antiangiogenic effects mediated by targeting of VEGF. Int J Cancer.

[63] Weissenberger J, Loeffler S, Kappeler A, Kopf M, Lukes A, Afanasieva TA (2004). IL-6 is required for glioma development in a mouse model. J Oncogene.

[64] Shi Y, Frost P, Hoang B, Benavides A, Gera J, Lichtenstein A (2011). IL-6-induced enhancement of c-Myc translation in multiple myeloma cells: critical role of cytoplasmic localization of the rna-binding protein hnRNP A1. J Biol Chem.

[65] Grivennikov S, Karin E, Terzic J, Mucida D, Yu GY, Vallabhapurapu S (2009). IL-6 and Stat3 are required for survival of intestinal epithelial cells and development of colitis-associated cancer. Cancer Cell.

[66] Garcia-Tuñón I, Ricote M, Ruiz A, Fraile B, Paniagua R, Royuela M (2005). IL-6, its receptors and its relationship with bcl-2 and bax proteins in infiltrating and in situ human breast carcinoma. Histopathology.

[67] Culig Z (2014). Proinflammatory cytokine interleukin-6 in prostate carcinogenesis. Am J Clin Exp Urol.

[68] von Felbert V, Córdoba F, Weissenberger J, Vallan C, Kato M, Nakashima I (2005). Interleukin-6 gene ablation in a transgenic mouse model of malignant skin melanoma. Am J Pathol.

[69] Yu F, Wang X, Guo ZS, Bartlett DL, Gottschalk SM, Song XT (2014). T-cell engager-armed oncolytic vaccinia virus significantly enhances antitumor therapy. Mol Ther.

[70] Contag CH, Sikorski R, Negrin RS, Schmidt T, Fan AC, Bachireddy P (2010). Definition of an enhanced immune cell therapy in mice that can target stem-like lymphoma cells. Cancer Res.

[71] Thorne SH (2014). Immunotherapeutic potential of oncolytic vaccinia virus. Front Oncol.

[72] Teigler JE, Phogat S, Franchini G, Hirsch VM, Michael NL, Barouch DH (2014). The canarypox virus vector ALVAC induces distinct cytokine responses compared to the vaccinia virus-based vectors MVA and NYVAC in rhesus monkeys. J Virol.

