# Erratum to: Oncolytic virus efficiency inhibited growth of tumour cells with multiple drug resistant phenotype in vivo and in vitro

**DOI:** 10.1186/s12967-016-1041-3

**Published:** 2016-09-27

**Authors:** Elena P. Goncharova, Julia S. Ruzhenkova, Ivan S. Petrov, Sergey N. Shchelkunov, Marina A. Zenkova

**Affiliations:** 1 Institute of Chemical Biology and Fundamental Medicine SB RAS, 8, Lavrentiev Ave., Novosibirsk, 630090 Russian Federation; 2 Institute of Cytology and Genetics SB RAS, Novosibirsk, Russian Federation; 3 Department of Biochemistry, Biocenter, University of Wuerzburg, Am Hubland, 97074 Würzburg, Germany

## Erratum to: J Transl Med (2016) 14:241 DOI 10.1186/s12967-016-1002-x

Unfortunately, the original version of this article [1] contained an error. Figures 2 and 7 were the incorrect versions. They have been corrected in the original article and are also included correctly in this erratum.

**Fig. 2:**
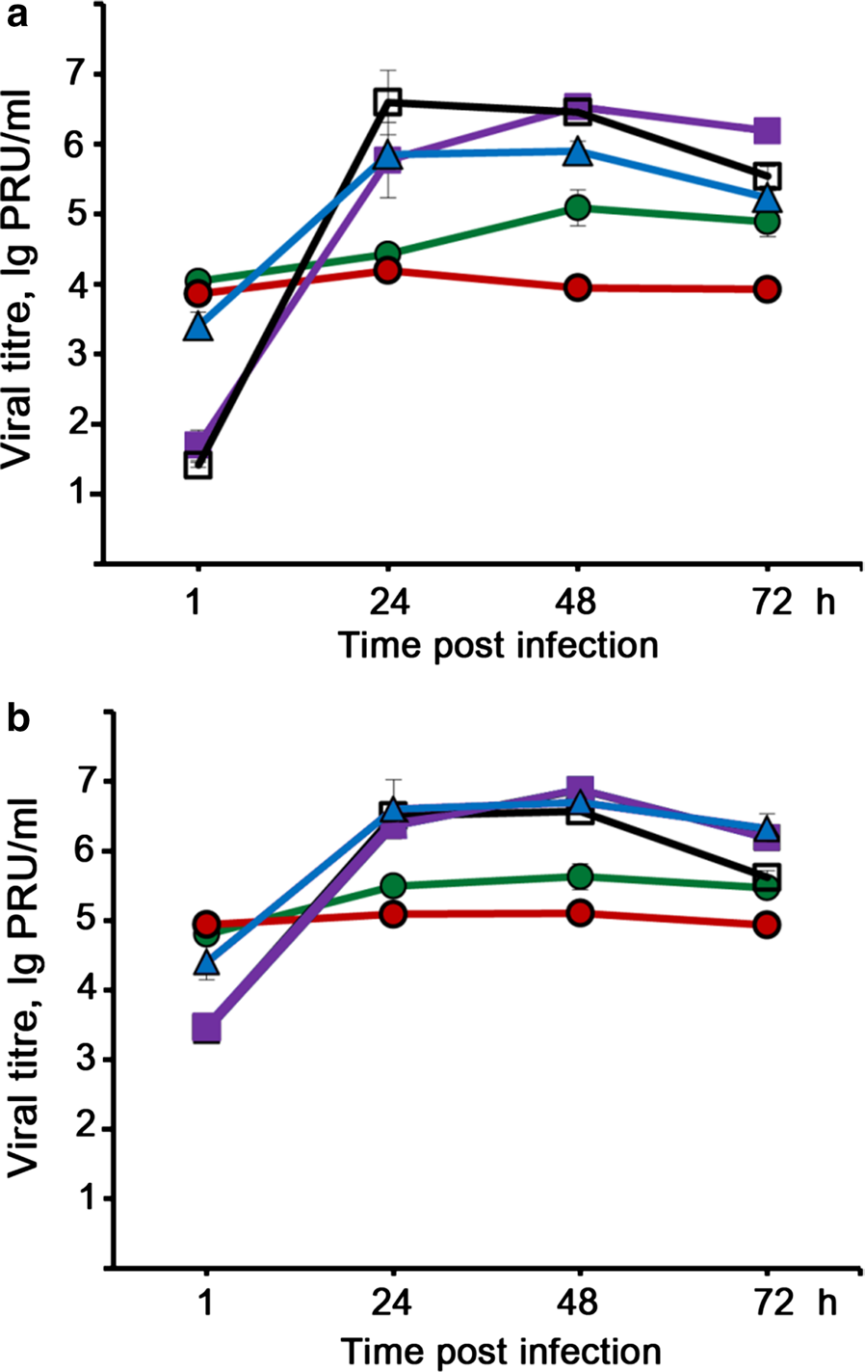
Development of LIVP-GFP infection in various tumour cells. Development of LIVP-GFP infection in RLS (*green circles*), RLS-40 (*red circles*), KB-3-1 (*violet squares*), KB-8-5 (*black line with open squares*) and melanoma B-16 (*blue triangles*) tumour cells at MOI of 1 (**a**) and MOI of 10 (**b**). Cells were incubated with virus for 1 h, washed with PBS and incubated up to the analysis in IMDM supplemented with 2 % FBS. Viral titre was measured by PFU assay. Data of three independent experiments are presented

**Fig. 7:**
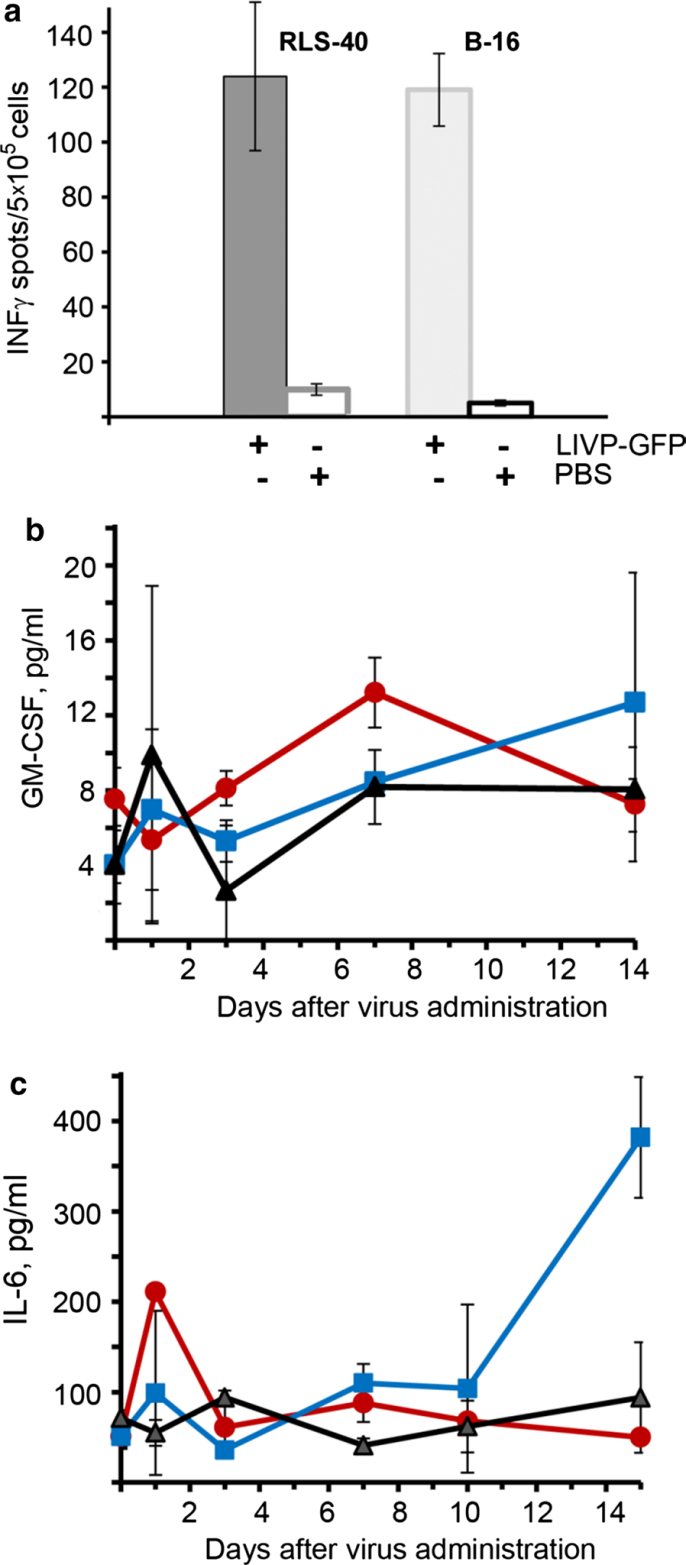
Effect of LIVP-GFP treatment on the immune responses of tumour-bearing mice. **a** Summary data showing a significant increase of the number of IFN-γ-ransecreting splenocytes in LIVP-GFP-treated mice (n = 6) with RLS-40 or with melanoma B-16 tumours. Comparison of immune-related proteins GMC-SF (**b**) and IL-6 (**c**) in the blood serum of RLS-40 bearing mice during the treatment with LIVP-GFP (the experimental scheme was shown in Fig. 5a): *red circles* and *blue squares* for RLS-40 bearing mice treated with LIVP-GFP and PBS, respectively; *black triangles* healthy mice receiving PBS. The levels of cytokines in the blood serum were measured by ELISA. For each day, the value of mean ± SEM is shown

## References

[1] Goncharova EP, Ruzhenkova JS, Petrov IS, Shchelkunov  SN, Zenkova  MA (2016). J Transl Med.

