# Supplementary material for: Oncolytic virus efficiency inhibited growth of tumour cells with multiple drug resistant phenotype in vivo and in vitro
Source: J Transl Med. 2016 Aug 18;14:241. doi: 10.1186/s12967-016-1002-x (PMC4989492; doi:10.1186/s12967-016-1002-x)
Supplement: Supplementary file 1 — 10.1186/s12967-016-1002-x The original flow cytometry images showing development of LIVP-GRF infection at MOI = 1 or MOI = 10 in different tumour cells 2, 24 and 48 hpi. Table S1. The effect of LIVP-GFP treatment on the liver of RLS-40-bearing mice. [file 12967_2016_1002_MOESM1_ESM.doc]

**Supplementary materials**

**Fig. S1.** The original flow cytometry images showing development of LIVP-GRF infection at MOI = 1 or MOI = 10 in different tumour cells 2, 24 and 48 hpi.

**Table S1.** The effect of LIVP-GFP treatment on the liver of RLS-40-bearing mice.

|  | Healthy СВА  (MEAN±SEM) | CВА mice with intramuscularly implanted RLS_40_ | |
| --- | --- | --- | --- |
|  |  | RLS_40_ + PBS ^a^ (MEAN±SEM) | RLS_40_ + LIVP-GFP (MEAN±SEM) |
| Normal liver parenchyma,  Vv ^c^, % | 76.9±1.5 | 36.7±3.7 * | 65.4±2.0 * # |
| Dystrophy, Vv ^c^, % | 7.3±0.7 | 22.4±2.6 * | 12.3±0.8 * # |
| Necrosis, Vv ^c^, % | 3.1±0.4 | 38.7±1.4 * | 17.3±1.4 * # |
| Total destructive changes,  Vv ^c^, % | 10.4±0.9 | 61.1±3.6 * | 29.6±2.2 * # |
| Binuclear hepatocytes, Nv ^d^ | 1.5±0.2 | 0.7±0.2 * | 0.7±0.1 |

* statistically significant difference relative to healthy СВА, p ≤ 0.05;

# statistically significant difference relative to the RLS_40_ + PBS group, p ≤ 0.05.

^a^ RLS_40_ + PBS - group of mice that received PBS injections.

^b^ RLS_40_ + LIVP-GFP - group of mice that received LIVP-GFP treatment.

^c^ The volume density (Vv) representing the volume fraction of tissue occupied by this compartment.

^d^ The numerical density (Nv) indicating the number of particles in the unit tissue volume.
